# Non-invasive Model-Based Assessment of Passive Left-Ventricular Myocardial Stiffness in Healthy Subjects and in Patients with Non-ischemic Dilated Cardiomyopathy

**DOI:** 10.1007/s10439-016-1721-4

**Published:** 2016-09-07

**Authors:** Myrianthi Hadjicharalambous, Liya Asner, Radomir Chabiniok, Eva Sammut, James Wong, Devis Peressutti, Eric Kerfoot, Andrew King, Jack Lee, Reza Razavi, Nicolas Smith, Gerald Carr-White, David Nordsletten

**Affiliations:** 1 Division of Imaging Sciences and Biomedical Engineering, King’s College London, St. Thomas’ Hospital, London, SE1 7EH UK; 2 Inria and Paris-Saclay University, Bâtiment Alan Turing, 1 rue Honoré d’Estienne d’Orves, Campus de l’Ecole Polytechnique, 91120 Palaiseau, France; 3 Department of Engineering Science, University of Auckland, 20 Symonds St, Auckland, 1010 New Zealand

**Keywords:** Stiffness, Myocardium, Patient-specific modelling, Model uncertainties, Parameter uniqueness

## Abstract

Patient-specific modelling has emerged as a tool for studying heart function, demonstrating the potential to provide non-invasive estimates of tissue passive stiffness. However, reliable use of model-derived stiffness requires sufficient model accuracy and unique estimation of model parameters. In this paper we present personalised models of cardiac mechanics, focusing on improving model accuracy, while ensuring unique parametrisation. The influence of principal model uncertainties on accuracy and parameter identifiability was systematically assessed in a group of patients with dilated cardiomyopathy ($n=3$) and healthy volunteers ($n=5$). For all cases, we examined three circumferentially symmetric fibre distributions and two epicardial boundary conditions. Our results demonstrated the ability of data-derived boundary conditions to improve model accuracy and highlighted the influence of the assumed fibre distribution on both model fidelity and stiffness estimates. The model personalisation pipeline—based strictly on non-invasive data—produced unique parameter estimates and satisfactory model errors for all cases, supporting the selected model assumptions. The thorough analysis performed enabled the comparison of passive parameters between volunteers and dilated cardiomyopathy patients, illustrating elevated stiffness in diseased hearts.

**Electronic supplementary material:**

The online version of this article (doi:10.1007/s10439-016-1721-4) contains supplementary material, which is available to authorized users.

## Introduction

With cardiovascular disease being the leading cause of death worldwide,^37^ significant research effort has been devoted to understanding heart function in health and pathology. As a wide range of aetiologies have been attributed to cardiac conditions, determining the factors influencing disease in individual patients—and selecting appropriate treatments—remains an ongoing challenge. In some cases, such as hypertrophic cardiomyopathy, myocardial infarction and diastolic heart failure, abnormalities in tissue stiffness have been identified as features of the disease.^36^ Structural alterations associated with the severity of the condition appear to be reflected in myocardial stiffness,^5^,^7^ suggesting its potential clinical utility in improving patient assessment and providing tailored treatment strategies.

Quantification of myocardial stiffness is not a straightforward task. Shear and stretch tests have been performed on animal and human tissue samples^9^,^32^ to provide a basis for estimation of myocardial properties.^16^,^30^ While the utility of these studies can hardly be overstated, the numerical values obtained from *ex vivo* data cannot necessarily be directly applicable in personalised *in vivo* studies. Alternatively, a number of techniques have been proposed that merge clinical data with mathematical models of varying complexity to obtain an indirect approximation to patient-specific myocardial properties. These range from established chamber stiffness estimates derived from pressure-volume curves^5^ or wall stress surrogates^1^ to developing detailed 2D or 3D strain estimates derived from Doppler echocardiography^10^,^19^ or magnetic resonance (MR) images^4^,^34^ to emerging wave propagation velocity estimates in shear wave ultrasound^8^,^15^ and MR elastography.^24^,^29^

Incorporating personalised geometries and loading conditions along with more physiologically accurate material responses, patient-specific modelling presents an alternative for stiffness estimation. In particular, data-derived information such as cavity volumes^7^ and pressures^3^,^13^ has been used to quantify model passive stiffness. The transition from bulk measures to more comprehensive data, such as tissue displacements and strains, has enabled more elaborate approaches for estimating a larger number of parameters^12^,^35^ and heterogeneous parameter distributions.^25^,^38^ As recent advances in medical imaging offer increasingly more detail on the heart anatomy and regional kinematics, rich datasets for model personalisation and characterisation of passive parameters are becoming more accessible.^2^,^14^,^39^

Despite the wealth of clinical data available and the continuous enhancements in the complexity and accuracy of personalised models, significant work is still required to enable the translation of model-based stiffness assessment to the clinic. A fundamental prerequisite is the reliable quantification of myocardial stiffness, an issue tightly coupled to the unique and accurate estimation of model parameters which was analysed in our previous study.^14^ Briefly, while extracting an error-minimising set of parameters is generally attainable, demonstrating that the obtained estimates are meaningful—and thus potentially clinically useful—requires ensuring parameter uniqueness. While previous studies have proposed a variety of techniques for obtaining unique parameter estimates,^13^,^38^ reliable parametrisation also depends on the model’s ability to accurately represent individual hearts (*model fidelity*), raising the core challenge of dealing with model uncertainties.^14^ Important modelling aspects such as the unstressed reference domain, the material law or appropriate boundary conditions remain relatively unknown, despite the wide range of experimental and modelling studies. Additionally, measurements such as Diffusion Tensor MRI (DTMRI) or cavity pressures which are valuable in model personalisation are often not part of routinely acquired clinical data, introducing further model uncertainties. Previous works have employed a variety of constitutive laws,^12^,^35^ rule-based fibre distributions^39^ and data-derived reference geometries^2^,^7^ to address these uncertainties; however, the suitability of the embedded model assumptions has not yet been systematically assessed in real data. Examining the influence of such assumptions on error—and hence model accuracy—illustrates potential improvements within a given model.

With these concerns in mind, in this work we develop patient-specific models of cardiac mechanics, focusing on the need for reliable parametrisation. The personalised models are built using comprehensive state-of-the-art clinical data [cine and 3D tagged MRI (TMRI)] which were non-invasively acquired. A reduced version of the Holzapfel–Ogden^16^ law is employed, in accordance with our previous works^2^,^14^ whereby it was identified as a suitable choice for parameter estimation applications based on TMRI. Important model uncertainties are systematically examined, with the objective of improving model accuracy while ensuring stiffness identifiability. In particular, we examine the influence of the fibre distribution on model fidelity, as fibre architecture is a key determinant of cardiac function. Epicardial boundary conditions are also investigated, as a means of incorporating data into the model and enhancing model fidelity. The robustness and suitability of the selected assumptions are assessed in healthy volunteers ($n=5$) and patients with dilated cardiomyopathy (DCM, $n=3$). Due to the ventricular remodelling observed in DCM hearts, passive parameters are expected to differ between the two groups,^7^,^18^,^27^,^28^ providing a framework for evaluating the proposed approach for stiffness assessment. Finally, having verified unique parametrisation, we compare parameters between volunteers and patients, enabling a preliminary assessment of variation in stiffness between healthy and DCM hearts.

Below, we expand on our approach to estimate and study myocardial stiffness through patient-specific modelling. Building on the data processing and model development protocol presented in our recent studies^2^,^14^ in “Materials and Methods” section, we review the process followed for the creation of personalised models. Modelling aspects that can influence model fidelity and parameter identifiability *in vivo* are examined in “Results” section, allowing comparisons between DCM patients and healthy volunteers. Our findings are reviewed and discussed in “Discussion” section, providing directions for future research.

## Materials and Methods

**Figure 1:**
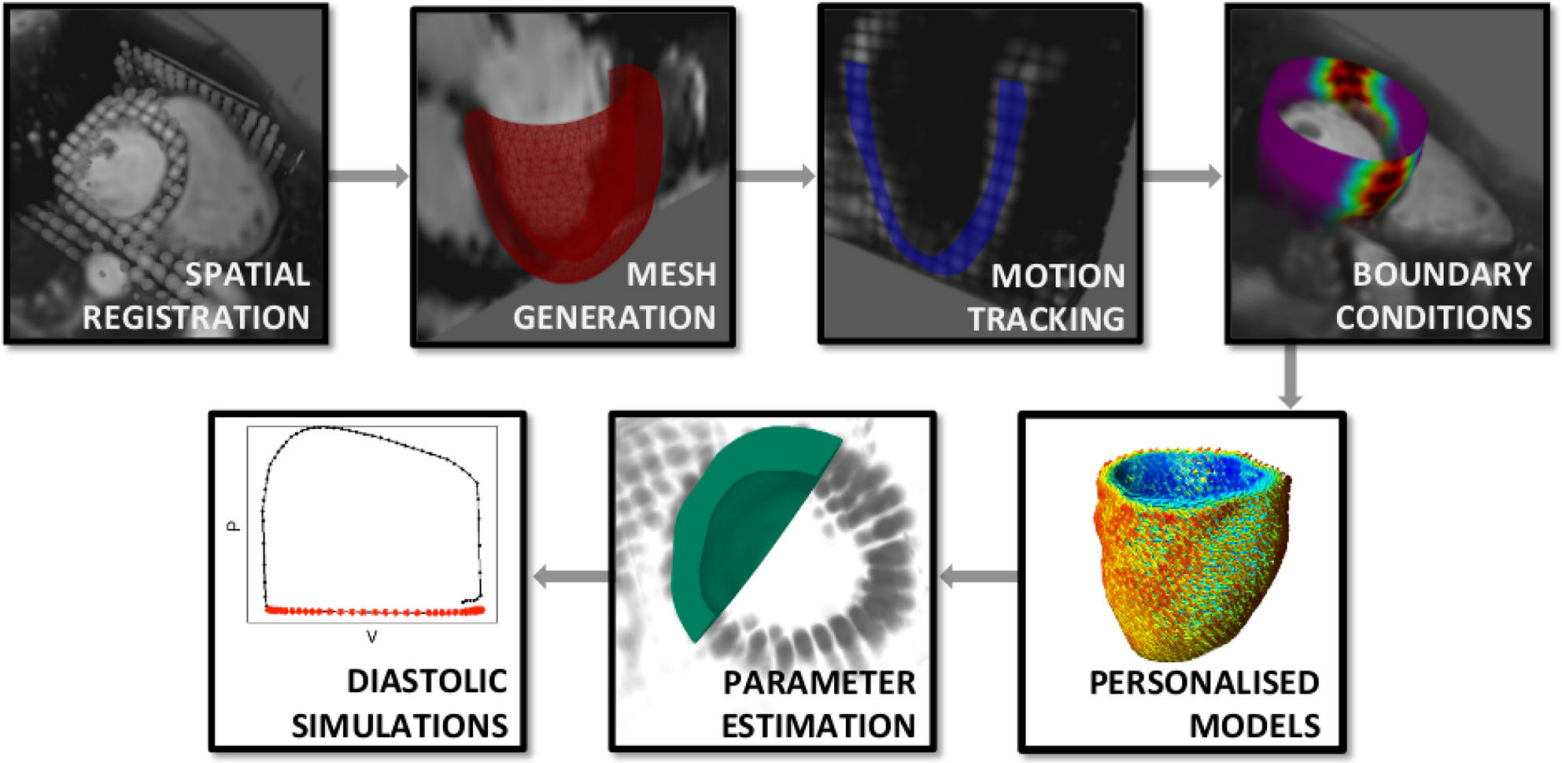
Workflow followed for the development and analysis of personalised diastolic heart models. Following spatial registration of images, segmentations of end-diastolic cine images were used to create an LV mesh, on which motion extracted from TMRI was propagated through the cardiac cycle. Personalised models were driven by extracted cavity volumes, while data-derived boundary conditions were applied on the basal and epicardial boundaries. Finally, parameter estimates were obtained through minimisation.

In this section we present the process followed for assessing stiffness using personalised diastolic left ventricular (LV) models of cardiac mechanics. The model personalisation pipeline, summarised in Fig. 1 1, is presented in “Data Processing Pipeline” section. The mechanics model is then reviewed along with the process for estimating model parameters (“Personalised Cardiac Mechanics Model” section). To assess the reliability of the obtained parameters we examine model uncertainties that can influence and improve parameter identifiability and model accuracy (“Model Fidelity and Parameter Identifiability Study” section).

**Table 1:** Participants’ general information and global LV indices.

Case	Age (years)	G	EDV (ml)	ESV (ml)	SV (ml)	EF	WT_ED_ (mm)	WT_ES_ (mm)	$\frac{{{\text{LA}}}}{{{\text{SA}}}}$	EDP^est^ (mmHg)
Volunteers										
V1	28	M	129.5	64.6	64.9	0.50	8.03	10.61	1.35	10.5
V2	29	F	100.9	47.7	53.2	0.53	7.09	9.07	1.59	16.1
V3	48	M	152.6	67.4	85.1	0.56	8.73	12.1	1.40	10.2
V4	35	F	93.2	46.0	47.2	0.51	6.58	8.74	1.59	8.8
V5	41	M	120.4	53.5	66.9	0.56	8.38	11.38	1.33	11.4
Mean	36.2	–	119.3	55.8	63.5	0.53	7.76	10.34	1.45	11.4
SD	8.4	–	23.6	9.7	14.6	0.03	0.90	1.45	0.13	2.8
DCM patients										
P1	28	F	141.1	81.9	59.2	0.42	7.92	10.11	1.23	16.3
P2	55	M	179.3	95.2	84.1	0.47	7.94	10.33	1.21	17.6
P3	43	F	136.2	79.9	56.3	0.41	6.54	8.24	1.24	11.6
Mean	42	–	152.2	85.7	66.5	0.43	7.46	9.56	1.22	15.2
SD	13.5	–	23.6	8.3	15.3	0.03	0.80	1.15	0.18	3.2

*G* gender, *EDV* end-diastolic volume, *ESV* end-systolic volume, *SV* stroke volume, *EF* ejection fraction, WT_ED_ and WT_ES_ wall thickness at end diastole and end systole, *LA*/*SA* ratio of long-axis to short-axis dimensions at end diastole, EDP^est^ estimated end-diastolic pressure. Metrics are based on TMRI processed motion and their computation is summarised in section S2 in Supplementary Material

### Data Processing Pipeline

Clinical data was collected from 5 volunteers with healthy heart function (V1–V5) and 3 DCM patients (P1–P3), recruited at St Thomas’ hospital. The study included male and female participants, with ages ranging from 28 to 55 (Table 1). Cardiac MRI scans (Table 2) were performed on a 1.5T Philips Achieva system. Written informed consent was obtained from all participants prior to scanning, and the protocol (study number 12/LO/1456) was approved by the London Bridge NRES committee.

**Table 2:** Basic information on the images acquired for all participants, in either prospective, *P*, or retrospective, *R*, ECG gating (ECG G).

Image type	ECG G	SR (mm)	TR (ms)
SA	R	2 × 2 × 8	20–30
2CH	R	2 × 2	20–30
3CH	R	2 × 2	20–30
4CH	R	2 × 2	20–30
TMRI	P	3 × 3 × 7 rec. 1 × 1 × 1	29–32
4D PCMRI	P	2.5 × 2.5 × 2.5	35–40

Images included cine MRI (in short-axis (SA) and long-axis 2-chamber (2CH), 3-chamber (3CH) and 4-chamber (4CH) views), TMRI and 4D Phase Contrast MRI (4D PCMRI). SR and TR denote spatial and temporal resolutions respectively. Both acquired and reconstructed (rec.) spatial resolutions of TMRI are presented

In order to enable consistent use of the available data, spatial registration of images was performed using the Image Registration Toolkit (IRTK)^2^. Image registration was essential to minimise misalignment between images caused by changes in patient’s position and breathing pattern. It is worth noting that inconsistencies between breath holds were decreased by using an MRI respiratory navigator during the acquisition of the SA stack.

LV meshes were created based on manual segmentations of end-diastolic cine images. Myocardial wall and cavity segmentations of SA, 2CH, 3CH and 4CH images were created using ITK-SNAP,^41^ and merged into one isotropic mask, which was further refined and smoothed. A template surface mesh based on a statistical atlas^17^ was then fitted to the mask and truncated at the base of the heart. Subsequently, the warped surface mesh was used to create a volumetric linear tetrahedral mesh using Cubit^3^ and TetMesh-GHS3D by Distene S.A.S./INRIA. Basic characteristics of LV meshes are presented in Table 3. All personalised meshes have a mesh size *h* of approximately 3–4 mm. The specific mesh size is sufficiently small to allow for accurate parameter estimation,^3^ yet sufficiently large to enable the small computational cost required for the large number of parameter sweeps performed.

**Table 3:** Here, LNodes and QNodes refer to the number of linear and quadratic nodes, respectively and *h* refers to the mesh size in mm.

Personalised LV meshes								
Case	V1	V2	V3	V4	V5	P1	P2	P3
Elements	17,153	18,896	8,038	6,787	7,795	10,740	17,047	10,731
LNodes	4,048	4,375	2,327	2,050	2,088	2,747	4,173	2,986
QNodes	27,589	30,068	14,580	12,657	13,499	18,104	28,012	19,025
*h * ± STD (*h*)	3.33 ± 0.53	2.98 ± 0.27	4.43 ± 1.09	3.88 ± 0.82	4.34 ± 0.34	3.90 ± 0.56	3.47 ± 0.53	3.45 ± 0.67
*q * ± STD (*q*)	0.81 ± 0.10	0.83 ± 0.09	0.73 ± 0.14	0.74 ± 0.12	0.86 ± 0.09	0.75 ± 0.12	0.82 ± 0.10	0.78 ± 0.13

Element quality *q* was computed as* q* = 3*r*, *r* being the ratio of inradius to circumradius. The mesh size was computed as $h = {\det (\varvec{S})^{1/3}}$, ${\varvec{S}}$ being the affine mapping between elements in mesh and the unit right tetrahedron

Myocardial wall motion was extracted from TMRI images using a non-rigid registration algorithm in IRTK.^31^ The registration algorithm which is based on optimisation of a similarity measure between images and free-form deformations was applied only on TMRI images. A myocardial mask was used during motion tracking to restrict the effect of surrounding tissues and organs. The extracted motion was applied onto the end-diastolic mesh, resulting in deformed meshes following the heart motion throughout the cardiac cycle. Extracted displacements were then processed to ensure conservation of myocardial volume (section S1 in supplement). Extracted and processed motion were compared against manually tracked landmark points (section S2), presenting satisfactory accuracy in both cases.

End-diastolic pressures were estimated based on a common clinical surrogate, the $E / E_{\mathrm{a}}$ ratio, proposed by Nagueh *et al.*,^26^ where *E* denotes the peak early diastolic flow velocity through the mitral valve and $E_{\mathrm{a}}$ denotes the early diastolic velocity of the mitral valve annulus ($E = 1.24 E / E_{\mathrm{a}} + 1.9$). *E* was measured from 4D PCMRI using GyroTools GTFlow^4^ and $E_{\mathrm{a}}$ velocity was estimated at the lateral basal region using the displacements extracted from TMRI.

The presented data processing steps were essential for model personalisation, but also enabled the derivation of important clinical metrics (Table 1), allowing for additional comparisons between patients and volunteers.

### Personalised Cardiac Mechanics Model

The mechanics of the personalised diastolic models were solved using the principle of stationary potential energy, following Asner *et al.* 2,3. Briefly, the myocardium is initially defined by the reference domain $\Omega _0 \subset {\mathbb {R}}^3$ and initial coordinates ${\varvec{X}} \in \Omega _0$. At time *t*, as the heart model deforms it is described by its physical domain $\Omega (t) \subset {\mathbb {R}}^3$, using the coordinates of its current position ${\varvec{x}}={\varvec{X}} + {\varvec{u}}$ (${\varvec{u}}$ denotes the displacement). For the models considered, the myocardium was assumed to be incompressible. The potential energy $\Pi$ of the myocardium can then be written as a sum of the myocardial internal and external energies^2^,^3^

1 $$\begin{aligned} \Pi ({\varvec{u}}, p, {\varvec{\lambda}}) = \Pi _{\mathrm{int}}({\varvec{u}}, p) + \Pi _{\mathrm{ext}}({\varvec{u}}, {\varvec{\lambda }}), \end{aligned}$$

where ${\varvec{u}}$, *p* and ${\varvec{\lambda }}$ denote displacement, hydrostatic pressure and boundary Lagrange multipliers respectively (see “Constitutive Law” and “Boundary Conditions” sections). The primary variables (${\varvec{u}}, p, {\varvec{\lambda }}$) are found as the saddle-point solution of the potential energy functional:

2 $$\begin{aligned} \Pi ({\varvec{u}}, p, {\varvec{\lambda }}) = \inf _{\varvec{v}\in U} \sup _{(q \times {\varvec{\mu }}) \in W\times \Lambda } \Pi ({\varvec{v}}, q, {\varvec{\mu }}), \end{aligned}$$

minimising the internal energy of the system, while ensuring enforcement of the incompressibility and boundary constraints. Here $U \times W \times \Lambda$ are appropriate function spaces.^2^,^3^

As no undeformed, unstressed state is encountered during the cardiac cycle, the reference geometry needs to be either estimated using an inverse method^23^ or assumed to be approximated by data. Due to challenges in estimating the reference domain, arising from its dependence on constitutive law, material parameters and boundary conditions,^2^ selecting a data frame as the reference domain is a common approach.^12^,^35^,^38^ For the personalised models considered, the end-systolic frame of the extracted TMRI motion was assumed as the reference geometry $\Omega _0$.

Furthermore, due to the absence of data on the fibre architecture of each participant’s heart, a rule-based fibre distribution was applied for all cases. A circumferentially symmetric fibre field was used, with the fibre angle varying linearly between $+\theta$ and $-\theta$ from endocardium to epicardium.^33^ Although $\theta =60^{\circ }$ is commonly used, three different fibre distributions were considered (i.e. $\theta = \{50^{\circ }, 60^{\circ }, 70^{\circ }\}$).

#### Constitutive Law

The internal energy $\Pi _{\mathrm{int}}$ of the myocardium is dependent on the material properties of the tissue and the model selected to describe them. The myocardial tissue was assumed to be a hyperelastic, incompressible material, for which the internal energy $\Pi _{\mathrm{int}}$ can be expressed with respect to a strain energy function $\Psi$, as

3 $$\begin{aligned} \Pi _{\mathrm{int}}({\varvec{u}},p) = \int _{\Omega _0} \Psi ({\varvec{u}}) + p(J-1) {\mathrm{d}V.} \end{aligned}$$

In this case *J* denotes the determinant of the deformation gradient ${\varvec{F}}= \nabla _{\varvec{X}}{\varvec{u}} + {\varvec{I}}$.

The choice of constitutive law was dictated by one of the basic objectives of this work, namely sufficient model fidelity and unique parametrisation. In previous works with synthetic and real TMRI,^2^,^14^ a reduced version of the structurally-based Holzapfel–Ogden^16^ model was shown to satisfy these requirements. Accordingly, the passive response of the myocardium was modelled as:

4 $$\begin{aligned} \Psi ({\varvec{u}})= \frac{a}{2b}\left( \exp [b(II_{F}-3)] -1 \right) + \frac{a_{\mathrm{f}}}{2b_{\mathrm{f}}}\left( \exp [b_{\mathrm{f}}(II_{F_{\mathrm{f}}}-1)^2] -1\right) , \end{aligned}$$

with isotropic *a*, *b* and fibre $a_{\mathrm{f}}$, $b_{\mathrm{f}}$ material parameters. Here $II_{F}= {\varvec{F}}: {\varvec{F}}$ denotes the second invariant of ${\varvec{F}}$. This constitutive law accounts for the different material properties along the fibre direction ${\varvec{{\mathrm {f}}}_0}$, by using an invariant associated with fibres ($II_{F_{\mathrm{f}}} = {\varvec{F}}{\varvec{\mathrm {f}_0}}: {\varvec{F}}{\varvec{\mathrm {f}_0}}$). To restrict the number of parameters and assist unique parametrisation, the exponents *b* and $b_{\mathrm{f}}$ were not estimated but instead kept constant. The values used ($b=5$, $b_{\mathrm{f}}=5$) were chosen to ensure a physiological (and pathological for DCM patients) pressure-volume response,^14^ based on the empirical curve of Klotz *et al.* 20 Although the estimates of *a* and $a_{\mathrm{f}}$ are dependent^14^ on the specific choice of *b* and $b_{\mathrm{f}}$, assigning the same values to the exponents across cases allows for reliable comparisons of *a* and $a_{\mathrm{f}}$ between cases.

#### Boundary Conditions

The external energy $\Pi _{\mathrm{ext}}$ of the LV models was comprised of the sum of external boundary-based energies, applied on the basal (*b*), endocardial ($\ell$) and epicardial (*e*) surfaces. Data-derived constraints were enforced using Lagrange multipliers ${\varvec{\lambda }}_k$, resulting in the following form for the external energy,

5 $$\begin{aligned} \Pi _{\mathrm{ext}}({\varvec{u}}, {\varvec{\lambda }}) = \sum _{k \in (b, \ell , e)} \Pi _{\mathrm{ext}}^k({\varvec{u}}, {\varvec{\lambda }}_k). \end{aligned}$$

Overcoming the need for invasive cavity pressure measurements—commonly prescribed on the endocardial boundary^7^,^35^—in this work diastolic simulations were driven using cavity volume which can be easily extracted from routinely acquired images. Volume inflation was achieved by ensuring that the mesh lumen volume was equal to the data-derived cavity volume at every time step, using the Lagrange multiplier approach, as described in Asner *et al.* 2,3

A data-derived constraint was also applied on the base plane, which was set to follow the TMRI extracted motion. Following Asner *et al.*,^2^,^3^ a relaxed basal boundary condition was employed to restrict the influence of noisy tracked data on the model and avoid pressure and stress peaks arising with strict enforcement of displacement constraints.

A common practice in cardiac patient-specific applications is to assume that the epicardial energy is negligible. This approach was also examined in this work and will be referred to as the “No-traction boundary condition” (NT BC). The right ventricle (RV) is likely, however, to exert a substantial force on the septal wall of the LV during filling. To account for this effect, a relaxed boundary condition on the epicardial boundary was also examined, which incorporated data-derived motion on the region of attachment to the RV wall (RV BC). The specific region was defined through a spatial field *H* varying smoothly between 0 and 1, marking the RV attachment points with 1. In this case, the following form was used for the epicardial boundary energy,

6 $$\begin{aligned} \Pi _{\mathrm{ext}}^{e}({\varvec{u}}, {\varvec{\lambda}}_{\mathrm{e}}) = \int _{\Gamma _0^e} {\varvec{\lambda }}_{\mathrm{e}} \cdot \left( ({\varvec{u}} - {\varvec{u}}_{\mathrm{d}})H - \frac{1}{2}\epsilon _{\mathrm{e}} {\varvec{\lambda}}_{\mathrm{e}} \right) \ {\mathrm{d}}A, \end{aligned}$$

where $\Gamma _0^e$ is the undeformed epicardial surface, ${\varvec{u}}_{\mathrm{d}}$ is the data displacement and ${\varvec{\lambda }}_{\mathrm{e}}$ is the introduced Lagrange multiplier on the epicardial boundary. Three different values were considered for the relaxation parameter ($\epsilon _{\mathrm{e}} = \{5\times 10^{-8}, 5\times 10^{-6}, 5\times 10^{-4}\}$) and the moderate value ($\epsilon _{\mathrm{e}} = 5\times 10^{-6}$) was found to provide satisfactory adherence to the data without introducing spurious stresses.

#### Numerical Solution

The personalised models were solved using the finite element method. Specifically, a quadratic-linear interpolation scheme was employed for the displacement and hydrostatic pressure variables, respectively. The base and epicardial multipliers were approximated with quadratic triangular elements while the endocardial multiplier was a scalar. All problems were solved using ${\varvec {\cal C\it }}$ *Heart*, a multi-physics finite element solver.^22^

#### Parameter Estimation

Estimation of material parameters (*a*, $a_{\mathrm{f}}$) in Eq. (4) was achieved by minimising the objective function ${\mathcal {J}}$, based on the $L^2(\Omega _0)$ norm of the relative error through time:

7 $$\begin{aligned} {\mathcal {J}}= \left( \frac{\sum \limits _{n=1}^{N} ||{\varvec{u}}^n - {\varvec{u}}^n_{\mathrm{d}}||^2}{\sum \limits _{n=1}^{N} || {\varvec{u}}^n_{\mathrm{d}}||^2} \right) ^{1/2}. \end{aligned}$$

Here ${\varvec{u}}_{\mathrm{d}}^n$ and ${\varvec{u}}^n$ are the data-derived and model-predicted displacements at timestep *n*, respectively, and *N* is the number of diastolic frames identified in the available TMRI data.

In volume-driven simulations, estimation of both *a* and $a_{\mathrm{f}}$ parameters is not feasible using solely displacement observations. Due to the linear parameter dependence of the reduced Holzapfel–Ogden law, scaling of both passive parameters only scales cavity pressure but does not affect the displacements outcome. Nevertheless, exploiting this property we can restrict the parameter space to only one unknown, the parameter ratio $\gamma = a/a_{\mathrm{f}}$. $\gamma$ was estimated through parameter sweeps, which—even though computationally costly—provide a characterisation of parameter identifiability. The parameter sweeps (23 values were considered for $\gamma$, ranging between 0.3 and 2) were performed by keeping the fibre parameter $a_{\mathrm{f}}^{\mathrm{sim}}$ constant and varying $a^{\mathrm{sim}}$. An arbitrary value ( $a_{\mathrm{f}}^{\mathrm{sim}} = 1000Pa$) was used for the fibre parameter over sweeps. As any scaling of $a_{\mathrm{f}}^{\mathrm{sim}}$ will proportionally scale $\lambda _{\ell }^{\rm ED}$, the absolute values of both parameters (*a*, $a_{\mathrm{f}}$) can then be computed by scaling by the ratio between estimated (EDP^est^) and simulated ($\lambda _{\ell }^{\rm ED}$) end-diastolic pressure as:

8 $$\begin{aligned} a = a^{\mathrm{sim}} \frac{{\rm EDP}^{\mathrm{est}}}{\lambda _{\ell }^{\rm ED}}= 1000 \gamma \frac{{\rm EDP}^{\mathrm{est}}}{\lambda _{\ell }^{\rm ED}}, \quad \quad \quad \quad a_{\mathrm{f}} = 1000\frac{{\rm EDP}^{\mathrm{est}}}{\lambda _{\ell }^{\rm ED}}. \end{aligned}$$

To enable more accurate parameter estimates using RV BC and $\theta =50^{\circ }$, parameter sweeps were augmented with values closer to the previously obtained $\gamma$ estimates.

### Model Fidelity and Parameter Identifiability Study

Despite the variety of clinical data available which enabled the model personalisation presented in “Data Processing Pipeline” and “Personalised Cardiac Mechanics Model” sections, certain modelling aspects could not be determined and needed to be based on literature studies. With the objective of achieving reliable parametrisation through improving model accuracy, model assumptions regarding fibre distribution, suitable boundary conditions and the reference state were carefully analysed. The pipeline was applied with perturbations of these assumptions on all volunteer and patient cases, to enable a systematic assessment of model uncertainties. Model accuracy and parameter identifiability were then examined through characterisation of the objective function ${\mathcal {J}}$ in Eq. (7) over the parameter space ($\gamma$). The values of ${\mathcal {J}}$, the relative displacement error, at the estimated parameter were used to assess model accuracy, while the overall behaviour of ${\mathcal {J}}$ provided a characterisation of parameter identifiability.

Three different fibre distributions were examined, as discussed in “Personalised Cardiac Mechanics Model” section, with the maximum angle $\theta = \{50^{\circ }, 60^{\circ }, 70^{\circ }\}$. The effect of the epicardial boundary condition on model fidelity and parameter identifiability was also assessed, by considering both NT BC and RV BC, as discussed in “Boundary Conditions” section. Additionally, different data frames were employed as reference state, and compared with respect to their influence on both accuracy and identifiability.

## Results

### Model Fidelity and Parameter Identifiability *In Vivo*

**Figure 2:**
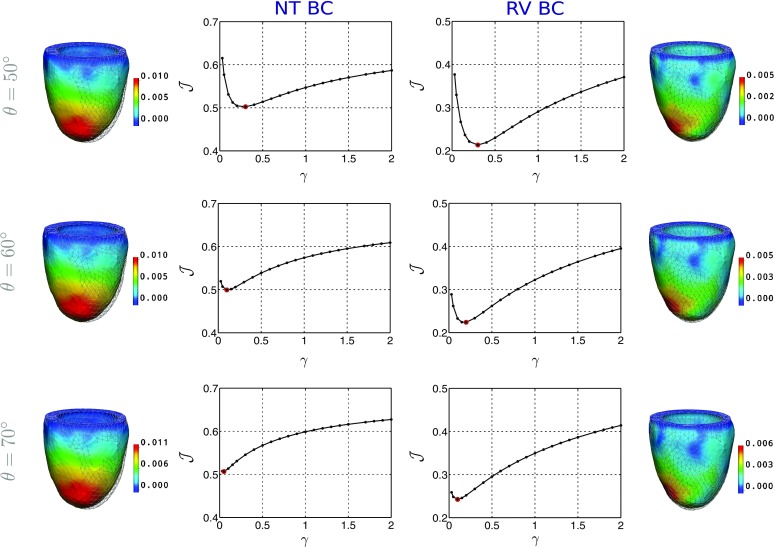
${\mathcal {J}}$ over the parameter ratio $\gamma$ for V5, for $\theta =\{{50^{\circ }, 60^{\circ }, 70^{\circ }}\}$ and NT BC / RV BC. Also presented are data-derived (mesh lines) and simulated (surface) end-diastolic states, with colour showing the error magnitude in metres.

Focusing on the need for reliable model parameters, this section examines model accuracy and parameter identifiability for all cases. Figure 2 illustrates the behaviour of ${\mathcal {J}}$ over the parameter ratio $\gamma$, for V5 (results for V1–V4 and P1–P3 are presented in supplementary material), with the three model fibre distributions and the NT BC / RV BC. Each data-point within these graphs represents a specific diastolic simulation compared to the tracked data. Additionally, Table S2 summarises $\gamma$ estimates for all cases.

**Figure 3:**
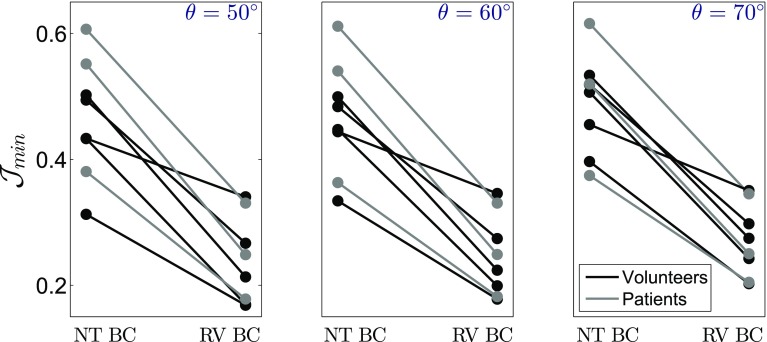
Model error (minimum value of the objective function) ${\mathcal {J}}_{\mathrm{min}}$ across fibre angle $\theta$, using NT BC and RV BC, for all the volunteers (in black) and DCM patients (in grey).

**Figure 4:**
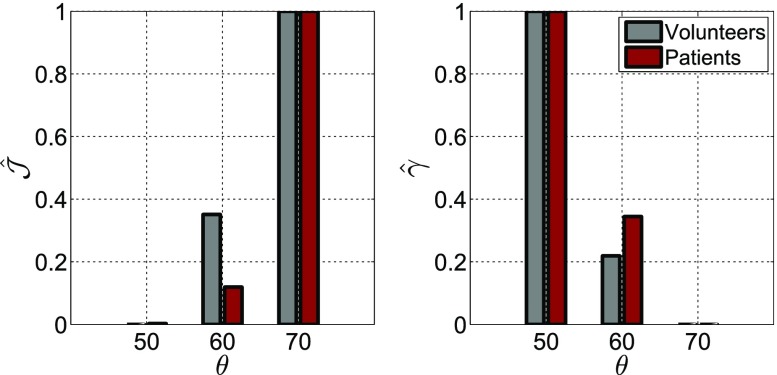
Effect of fibre distribution on (left) model error and (right) parameter ratio estimates, when the RV BC is employed. Here, normalised magnitudes are used for the objective function and parameter ratio estimates ($\hat{f} = \frac{f(\theta )-\min f(\theta )}{\max f(\theta ) -\min f(\theta )}$) and bars show average values over the volunteer and patient groups.

The effect of the employed epicardial boundary condition on all models is summarised in Fig. 3, which presents the change in model error. Similarly, Fig. 4 illustrates the influence of the fibre angle $\theta$ on model error and parameter estimates, in both volunteers and patients. Normalised values were used for both the objective function and parameter ratio estimates, to allow for comparison of the fibre angle effect between cases with different error/parameter ratio magnitudes.

**Figure 5:**
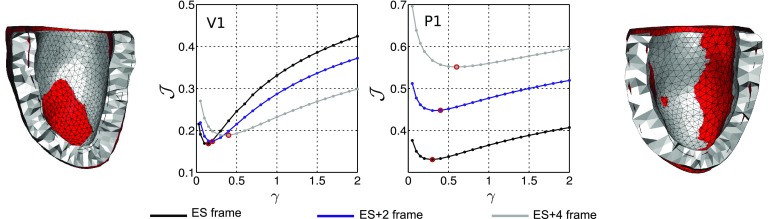
Objective function ${\mathcal {J}}$ over the parameter ratio $\gamma$, with three different data frames (end-systolic (ES), the second and fourth after ES) assumed as the reference, for (left) V1 and (*right*) P1. Bisected meshes present ES (grey) and ES+4 (red) geometries.

To assess the influence of the reference state, different diastolic frames were considered as reference $\Omega _0$ and the change in the behaviour of the objective function was investigated. Figure 5 compares ${\mathcal {J}}$ when different diastolic frames (end-systolic, second and fourth after end-systolic) were used, for a volunteer and a patient case (results for remaining cases are presented in the Supplementary Material).

### Comparative Analysis of *In Vivo* Cases

**Figure 6:**
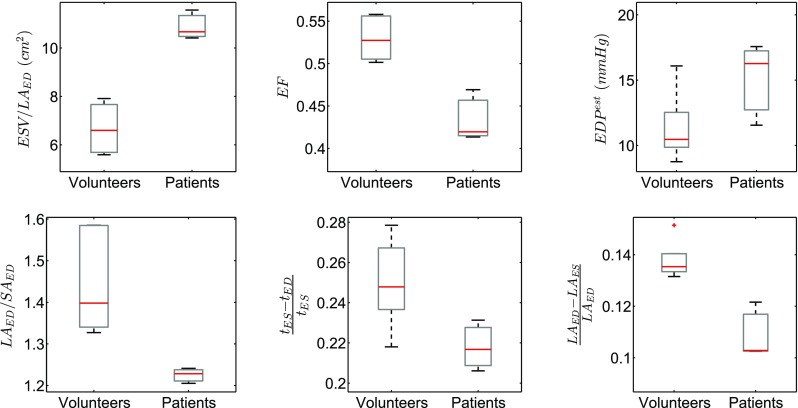
Comparison of data-derived metrics between the volunteer and patient groups. Red lines show the median, the boxes’ edges denote 25th and 75th percentiles, while black lines show extreme data points.

Following the results in “Model Fidelity and Parameter Identifiability *In Vivo*” section, a comparative analysis between volunteers and DCM patients was performed. Differences in clinical metrics (Table 1) between DCM and normals were analysed and unpaired *t*-tests were performed to assess their statistical significance. Variations in important metrics between the two groups are illustrated in Fig. 6. We note that reported cavity volumes differ from standard clinical volume measures due to the truncation of the LV mesh at a plane lower than the valve plane. As the truncation of the LV meshes cannot be easily standardised, volumes in Fig. 6 were normalised by the end-diastolic long-axis length to avoid biased comparisons.

**Figure 7:**
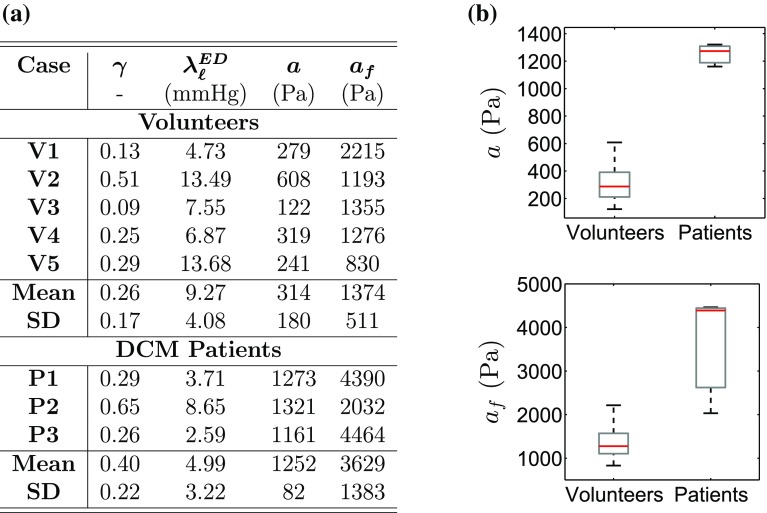
(*a*) Parameter estimates ($\gamma$, *a* and $a_{\mathrm{f}}$) for the volunteers and patients, along with the simulated end-diastolic pressure, $\lambda _{\ell }^{\rm ED}$. (*b*) Comparison of (top) isotropic parameter *a* and (bottom) fibre parameter $a_{\mathrm{f}}$ between the volunteer and patient groups.

Based on the analysis in “Model Fidelity and Parameter Identifiability *In Vivo*” section, model results were tabulated for RV BC and $\theta = 50^{\circ }$ for all cases. Parameter estimates are presented in Fig. 7, along with a schematic comparison of passive parameters between volunteers and patients.

## Discussion

### Model Fidelity and Parameter Identifiability

The ability of the model to accurately represent the mechanical behaviour of individual hearts is a prerequisite for the reliable estimation of stiffness with patient-specific modelling. Based on the tests presented, the boundary conditions and the employed fibre distribution were key factors in model accuracy.

In particular, observing the values of the objective function in Figs. 2 and 3, the application of the data-derived motion on the region of attachment to the RV proved superior compared to the simplified zero-traction epicardial boundary condition. In fact, the average model error decreased from approximately $\sim45$ to $\sim25\%$ and from $\sim51$ to $\sim26\%$, in the volunteer and patient groups respectively. The significant error reduction is also qualitatively illustrated in meshes in Fig. 2.

The application of RV BC also resulted in better identifiability characteristics as can be observed in Fig. 2. Specifically, the variation in ${\mathcal {J}}$ (difference between maximum and minimum ${\mathcal {J}}$ values) increased with RV BC, in accordance with the fact that sufficient model fidelity is a requirement for parameter identifiability.^14^ It is worth mentioning that the identifiability characteristics were also dependent on the magnitude of deformation, with a substantial deformation required for parameter identifiability. Accordingly, parameter identifiability was in general better in the healthy volunteers compared to DCM patients, where the cardiac deformation is lower.

The employed fibre architecture was also shown to notably impact model behaviour and parameter estimates. Firstly, based on Table S2 and Fig. 4, parameter ratio estimates were strongly coupled to the assumed fibre distribution. As the fibre angle increases we observe a greater trend toward anisotropy (*e.g.* $\gamma$ decreases). Furthermore, in the majority of cases employing the RV BC, the lowest error was obtained with a fibre angle of $\theta = 50^{\circ }$. This observation holds for all volunteer cases with a mean difference of $4.2 \%$ in ${\mathcal {J}}$, between $\theta = 50^{\circ }$ and $\theta = 70^{\circ }$. Although $\theta = 50^{\circ }$ also produced on average lower errors in the DCM group, the difference in error between $\theta = 50^{\circ }$ and $\theta = 70^{\circ }$ was less pronounced compared to the observed changes in the volunteer group. This observation suggests that angles could be steeper for DCM with minimal effect on the model. This would be in agreement with recent DTMRI studies,^11^ suggesting increased fibre angle in DCM.

Restricting the study to models employing the RV BC and a fibre distribution of $\theta = 50^{\circ }$, we can observe good identifiability characteristics for all cases. The presence of a clear minimum was combined with reasonable errors (17–34$\%$ and 18–33$\%$, for volunteer and patient groups, respectively), especially considering the strict metric used for ${\mathcal {J}}$ (relative $L^2$ error norm, accumulated over time). A good agreement between the model and the data was also presented in their average nodal distance in Table S3. With data varying notably in quality and consistency, the satisfactory errors observed not only suggest the potential of model assumptions but also illustrate the robustness of the model personalisation pipeline. Complementary evaluation of model accuracy was provided through regional strain distributions (Fig. S7 in the Supplementary Material) which demonstrated marked similarities between data and models.

Changing the reference frame between early-mid diastolic frames did not appear to affect parameter identifiability, with a unique minimum present for all reference geometries and in all cases. However, the parameter estimates were dependent on the frame employed. In particular, a consistent behaviour was observed for all volunteer cases, with the parameter ratio increasing when later data-frames were assumed as the reference domain. Similar behaviour was observed in the patient group, with two patients demonstrating increase in $\gamma$ and P3 presenting a small decrease in $\gamma$ when later frames were employed. Although the parameters were dependent on the frame selected as reference, the consistency in behaviour between cases in Figs. 5 and S6 suggests that the choice of reference state may introduce a consistent bias in the results, maintaining the ability for reliable comparisons between cases.

### Comparative Analysis of *In Vivo* Models

Despite the small number of volunteers and patients, distinct differences were identified in data-derived metrics between the two groups (Table 1; Fig. 6). Pronounced increases ($p \le 0.05$) were observed in end-diastolic and end-systolic cavity volumes in DCM hearts, as extensively reported in literature.^1^,^40^ Ejection fraction was considerably lower in the patient group ($p \le 0.005$), marking the deteriorated contractile and diastolic filling function typically clinically associated with DCM. Additionally, the decrease in the ratio of long-axis to short-axis dimensions observed in patients is characteristic of the change in shape from elliptical to spherical in DCM.^21^ Wall thickness was moderately decreased in DCM patients compared to normals both at end diastole and end systole, in accordance to other studies.^27^ A more evident trend was observed in the wall thickening ratio $(t_{\rm ES} - t_{\rm ED})/t_{\rm ES}$ ($p = 0.07$) and the normalised long-axis shortening $(LA_{\rm ED}- LA_{\rm ES})/LA_{\rm ED}$ ($p \le 0.005$), with both quantities decreasing in the DCM group. Similar findings have been reported in the literature,^40^ indicating reduced wall thickening during contraction and thus impaired contractile function in the presence of DCM. Analogous conclusions were also drawn from the magnitude of deformation (Table S3) as well as from regional strain distributions (Table S4) which indicated decreased strain metrics in DCM hearts compared to volunteers. Another key distinction was observed in the estimated end-diastolic pressure (EDP^est^). In accordance with previous studies,^1^,^21^ EDP^est^ was higher in the DCM group compared to normals, although this difference did not reach statistical significance ($p = 0.13$). The increased end-diastolic pressure is potentially linked to the impaired motion and lower strains observed in DCM hearts, with the end-diastolic pressure contributing to the deterioration of the tissue function and structure due to growth and remodelling.

Observing the obtained parameter ratios in Fig. 7a, it is worth noting that $\gamma$ values were significantly lower than 1. This finding is in agreement with experimental and modelling studies^35^ reporting that the myocardial tissue is stiffer in the fibre direction. Interestingly, $\gamma$—a measure of the degree of anisotropy—varied notably between cases, ranging between 0.09 and 0.65. With no significant difference in $\gamma$ identified between the volunteer and patient groups, it is possible that the degree of anisotropy is not markedly affected by the remodelling occurring in DCM hearts. However, as $\gamma$ is dependent on the fibre distribution (Fig. 4), the observed variation in $\gamma$ could also reflect differences in the fibre architecture amongst cases.

The differences were more pronounced in the absolute values of *a* and $a_{\mathrm{f}}$ (Fig. 7). Specifically, both the isotropic and fibre parameters *a* and $a_{\mathrm{f}}$ were distinctly higher in the DCM group compared to normals ($p = 0.0002$ and $p = 0.014$ respectively). The increase in the two parameter values indicates increased stiffness in DCM models.

A link between DCM and elevated stiffness has been reported for genetically engineered mice^7^,^27^ and humans^5^ based on pressure-volume analysis and patient-specific modelling.^5^,^27^ Increased stiffness appeared to be related to structural changes such as increase in the collagen content in the extracellular matrix,^6^,^18^ alterations in laminar architecture^27^ and presence of fibrosis,^5^ which could suggest potential treatment avenues. Additionally, a larger increase in stiffness was seen to reflect a later stage in the disease progression,^5^,^7^ supporting the potential use of stiffness as a clinical index for DCM assessment. In accordance with these findings, our results suggest increased stiffness in DCM hearts based on more comprehensive data and regional metrics. More importantly, the observation of elevated stiffness was based on a carefully selected model personalisation process focusing on unique parametrisation, providing confidence in the parameter estimates and conclusions.

#### Study Limitations

It should be noted, that parameter estimates can only suggest increased stiffness within the modelling framework employed, as model assumptions are likely to introduce bias into the estimated values. Future work should therefore be directed towards improving model accuracy, by addressing remaining model uncertainties. For instance, as fibre distribution was seen to significantly impact model outcomes, using *in vivo* DTMRI data or considering non-symmetric or nonlinear fibre distributions might prove beneficial for model fidelity. Additionally, the RV BC could be augmented to also account for the influence of the RV cavity pressure. Due to the absence of invasive RV cavity pressure or a reliable data-derived estimate, a simpler epicardial condition was used. Model fidelity and parameter accuracy could also be enhanced by allowing for regional variation in parameters and considering orthotropic material laws. Further improvement in model accuracy could be achieved by estimating the reference configuration through an inverse approach,^13^ for which, however, invasive pressure measurements would be necessary. Finally, important model uncertainties could be clarified over model validation, which would however require a large variety of invasive *in vivo* and *ex vivo* data.

Additionally, the accuracy of the model-derived stiffness could be affected by data-related factors. As parameter estimates are linearly dependent on EDP^est^ (Eq. 8), any error in the pressure estimation would be directly propagated to the parameter estimates. Parameter accuracy could thus be improved using pressure measurements, which are however invasive. Hence a non-invasive alternative was used that was verified over healthy volunteers and DCM patients.^26^ Furthermore, the resolution and noise in TMRI and cine MRI images as well as the accuracy of personalised segmentations could influence model outcomes. Use of more automated segmentation procedures and uncertainty quantification for model geometry will become increasingly necessary as models move closer to clinical translation. Additional error could be incorporated through the motion extraction algorithm that would propagate on both displacement and volume data that are key inputs for this work. Although the processed extracted motion demonstrated low errors against manually tracked landmarks, future work could be directed towards improving the agreement between processed data and original images for example by considering different boundary constraints over the incorporation of the wall-volume constraint (Eq. S2). Nevertheless, the obtained parameter estimates with and without the current post-processing step highlight the same distinction between healthy volunteers and DCM patients (Figs. 7b, S2).

The presented analysis was focused on diastole, therefore examining model uncertainties over whole cycle tests would provide a useful next step. As unique parametrisation has been established, additional tests can be performed with increased computational efficiency using data assimilation techniques. Furthermore, development of more personalised models for both healthy volunteers and DCM patients would enable a more thorough statistical analysis potentially strengthening our conclusions.

Finally, an interesting future development would be the use of data-based machine learning algorithms, which—provided a large enough cohort—could approximate the physical link between motion and stiffness that is now given by the model. However, such an endeavour would require a significant number of patients to account for non-trivial variabilities in structure and function.

## Conclusions

In this work we have collected comprehensive non-invasive clinical data from a group of healthy volunteers and DCM patients. Following data processing, metrics reported in the clinical literature were compared between the two groups, demonstrating significant deterioration in function in diseased hearts. Additional insight into cardiac function in DCM was obtained through patient-specific modelling, which enabled comparisons to transition from image-derived metrics to a model-based tissue stiffness. Acknowledging the need for reliable estimation of model stiffness, personalised models were developed with the objective of achieving sufficient model accuracy and unique parametrisation. Accordingly, key model uncertainties were systematically examined, elucidating that accounting for the mechanical influence of the RV and using a fibre angle of $\theta = 50 ^{\circ }$ produced consistently lower model errors and resulted in better identifiability characteristics. In fact, the assumed fibre distribution was shown to significantly influence model outcomes, highlighting the need for personalised fibre geometries. For all cases, unique parametrisation was combined with good agreement between model and data, supporting the adequacy of the employed model assumptions and the data processing followed. Passive stiffness was markedly higher in DCM models, suggesting that future research could be directed towards understanding its role in the onset and progression of the disease.

## Electronic supplementary material

Below is the link to the electronic supplementary material.

Supplementary material 1 (PDF 2,896 kb)

## Footnotes

1 Images were created using Eidolon (https://github.com/ericspod/Eidolon), developed by Dr Eric Kerfoot at King’s College London.

2
http://www.doc.ic.ac.uk/dr/software.

3
https://cubit.sandia.gov/.

4
http://www.gyrotools.com/products/gt-flow.html.
