# Supplementary material for: Non-invasive Model-Based Assessment of Passive Left-Ventricular Myocardial Stiffness in Healthy Subjects and in Patients with Non-ischemic Dilated Cardiomyopathy
Source: Ann Biomed Eng. 2016 Sep 7;45(3):605–18. doi: 10.1007/s10439-016-1721-4 (PMC5479360; doi:10.1007/s10439-016-1721-4)
Supplement: Supplementary file 1 — Supplementary material 1 (PDF 2,896 kb) [file 10439_2016_1721_MOESM1_ESM.pdf]

Supplementary Material

S1 Extracted motion processing

Preliminary analysis of the TMRI extracted motion showed large variation in myocardial wall volume over the cardiac cycle. Even though experimental results support that the wall volume is expected to vary by no more than 10%, in the preliminary tests it was reduced by as much as 30% at systole compared to end diastole, similar to the reduction observed in Shi *et al.*[1]. This significant variation in wall volume was considered non-physiological and was likely an artefact of the tracking algorithm, which did not ensure displacements were volume conserving. Additionally, the wall thickening over systole was significantly below physiological values for all cases (Table S1).

**Case**	**V1**	**V2**	**V3**	**V4**	**V5**	**P1**	**P2**	**P3**
$\mathbf{WT_{ED}}$	8.03	7.09	8.73	6.58	8.34	7.92	7.94	6.54
$\mathbf{WT_{ES}}$ **(Unprocessed)**	8.58	7.53	9.29	6.79	9.34	8.51	8.67	6.93
$\mathbf{WT_{ES}}$ **(Processed)**	10.61	9.07	12.09	8.74	11.38	10.11	10.33	8.24

Table S1 Wall thickness at end-diastole ($\mathrm{WT_{ED}}$) and end-systole ($\mathrm{WT_{ES}}$) for all cases. Both the unprocessed and processed end-systolic wall thicknesses are presented.

Accordingly, the motion tracked deformation field was modified to ensure that wall volumes were kept constant throughout the cycle and equal to the wall volume at end diastole. In order to ensure a close match to the original data, at each time-step the processed displacement field ($\boldsymbol{u}_d$) was obtained through a volume preserving H^1 projection of the original tracking data ($\boldsymbol{u}_o$). In this process, the endocardial boundary was kept weakly the same through a Lagrange multiplier $\boldsymbol{\lambda}_\ell$, and a relaxed boundary condition was introduced on the

base boundary[3]. The volume-preserving displacement field $\boldsymbol{u}_d$ was obtained by the solution of the following saddle-point problem:

$$\Pi(\boldsymbol{u}_d, \lambda, \boldsymbol{\lambda}_\ell, \boldsymbol{\lambda}_b) = \inf_{\boldsymbol{v} \in \boldsymbol{U}} \sup_{q \in W} \sup_{\boldsymbol{\mu}_\ell \in \Lambda_1} \sup_{\boldsymbol{\mu}_b \in \Lambda_2} \Pi(\boldsymbol{v}, q, \boldsymbol{\mu}_\ell, \boldsymbol{\mu}_b), \tag{S1}$$

where the functional Π at any point in time is given by:

$$\begin{aligned}\Pi(\boldsymbol{u}_d, \lambda, \boldsymbol{\lambda}_\ell, \boldsymbol{\lambda}_b) = &\int_{\Omega_{ED}} |\boldsymbol{u}_d - \boldsymbol{u}_o|^2 + \alpha|\nabla(\boldsymbol{u}_d - \boldsymbol{u}_o)|^2 dV + \int_{\Gamma^\ell_{ED}} \boldsymbol{\lambda}_\ell \cdot \big(\boldsymbol{u}_d - \boldsymbol{u}_o\big)\, dA \\ &+ \int_{\Gamma^b_{ED}} \boldsymbol{\lambda}_b \cdot \big(\boldsymbol{u}_d - \boldsymbol{u}_o - \frac{1}{2}\epsilon_b \boldsymbol{\lambda}_b\big)\, dA + \int_{\Omega_{ED}} \lambda(J_d - 1)\, dV.\end{aligned} \tag{S2}$$

The first integral term deals with the data projection, and $\alpha = 100$ was selected to provide equal weighting between the L^2 norm and H^1 semi-norm terms, over simulations in millimetres. The second and third terms are enforcing the original data motion on endocardial and basal boundaries, respectively. The final term is enforcing the incompressible deformation using Lagrange multiplier λ, where J_d is the determinant of the deformation gradient related to the projected displacement $\boldsymbol{u}_d$. Here, Ω_{ED} is the myocardial wall domain at end diastole, and Γ^ℓ_{ED} and Γ^b_{ED} denote the endocardial and basal boundary domains at end diastole, respectively. The employed boundary conditions were selected to ensure consistency between processed and unprocessed quantities of interest. In particular, the endocardial boundary displacements were enforced from the original extracted motion, ensuring that metrics such as stroke volume and ejection fraction were maintained.

The resulting processed meshes were compared against images, demonstrating good agreement over the cardiac cycle as illustrated in a representative example in Figure S1. Interestingly, the post-processing step enabled physiological wall thickening during systole (Table S1). Additionally, parameter identifiability and reasonable model errors were observed using both processed and unprocessed data. While modest differences were observed in the obtained parameters (relative change up to 20% for a and up to 40% for a_f) the variation between volunteers and patients is consistent (Figures 7b and S2), suggesting that the volume preserving processing step did not fundamentally alter model-derived stiffness.

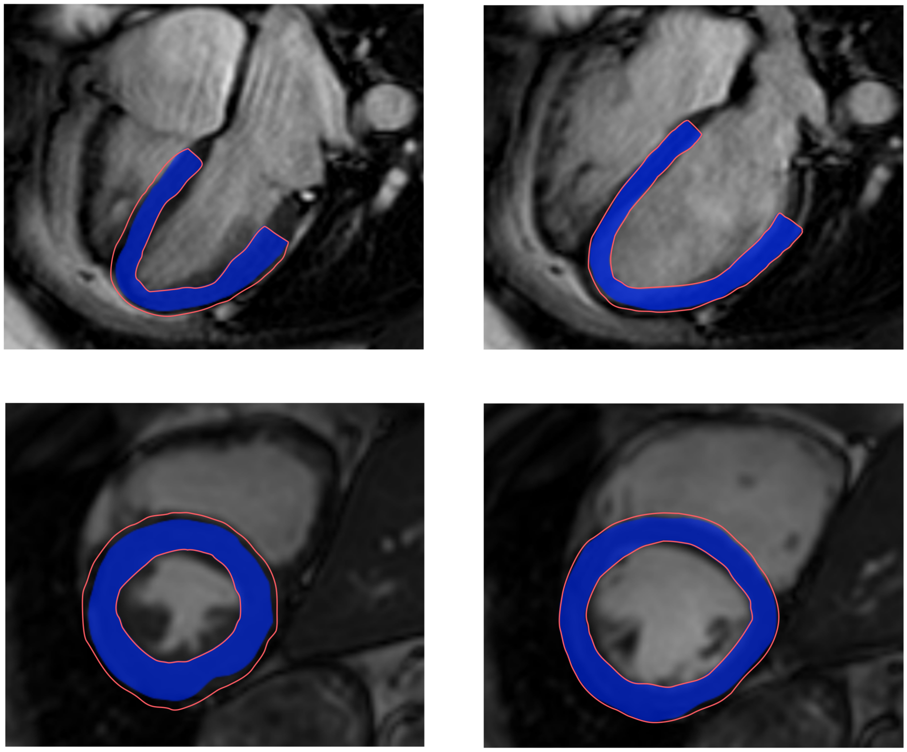

Figure S1 Unprocessed (blue mesh) and processed (red) extracted motion at end-systole (left) and end diastole (right), compared against cine images in long-axis and short-axis views, for V5.

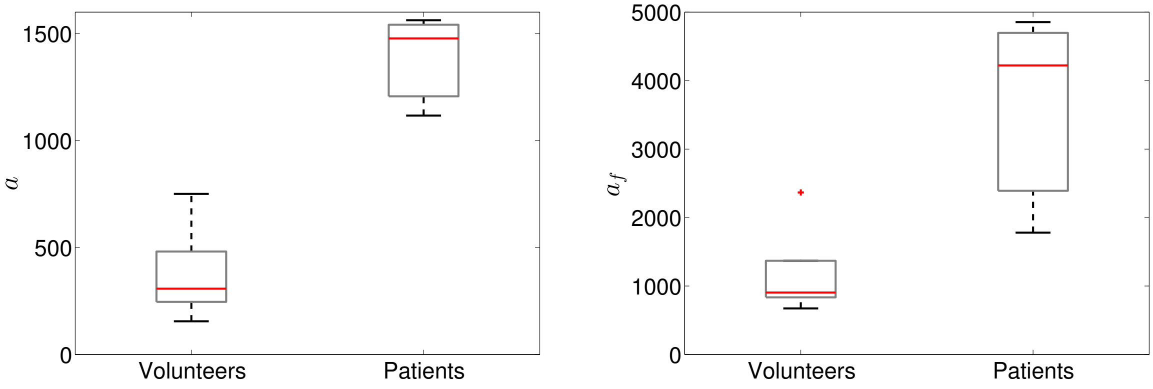

(a) **(b)**

Figure S2 Comparison of **(a)** isotropic parameter a and **(b)** fibre parameter a_f between the volunteer and patient groups, when the unprocessed extracted motion was used in model personalisation.

S2 Evaluation of extracted / processed motion accuracy

The accuracy of both the extracted and processed motion was evaluated against manually tracked points, following the methodology described in Shi *et al.*[1]. In particular, for each case 16 landmark points were selected, lying mid wall in each AHA region[2]. The position of the landmark points was manually tracked through the cycle, $\boldsymbol{p}^{AHA}_{manual}(t)$, along with their displacement, $\boldsymbol{u}^{AHA}_{manual}(t)$. The relative tracking error $\rho^{AHA}(t)$ for each AHA landmark point through the cardiac cycle was assessed as proposed in Shi *et al.*[1]:

$$\rho^{AHA}(t) = \frac{||\boldsymbol{p}^{AHA}_{manual}(t) - \boldsymbol{p}^{AHA}_{tracked}(t)||}{\max\limits_{s\in[0,T]} ||\boldsymbol{u}^{AHA}_{manual}(s)||}. \tag{S3}$$

Here $\boldsymbol{p}^{AHA}_{tracked}(t)$ refers to the position of the landmark point, based on the IRTK algorithm or the processed extracted motion.

Figure S3a presents the average tracking error over all cases, for both the IRTK extracted motion and the processed motion. These results show that the relative tracking errors for the IRTK extracted motion are consistent with Shi *et al.*[1]. Additionally, for a volunteer case manual tracking was performed by two individuals, showing that the error between the two operators ($\sim 0.08 \pm 0.02$) was close to the error between manual tracking and IRTK tracking.

Across the tests considered, it was observed that the error metric in Eq. S3 tends to be biased by relative errors in regions with reduced motion. This is due to the fact that the metric expects precision proportional to the motion observed in each AHA segment. Regions with little motion introduce much larger relative errors (Figure S3b), leading to an increase in the mean errors. However, for the purposes of this work we are more concerned with the overall motion than relative motion when comparing to the personalised models. Therefore, we have additionally estimated tracking errors using a maximal displacement metric:

$$\hat{\rho}^{AHA}(t) = \frac{||\boldsymbol{p}^{AHA}_{manual}(t) - \boldsymbol{p}^{AHA}_{tracked}(t)||}{||\boldsymbol{u}^{max}_{manual}||}. \tag{S4}$$

In this case, the error is divided by the maximum displacement over all AHA regions $\boldsymbol{u}^{max}_{manual}$,

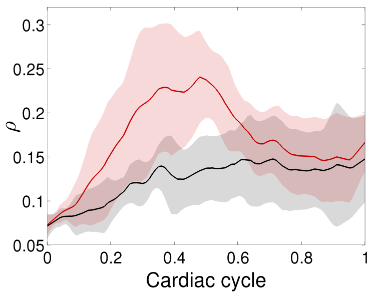

(a)

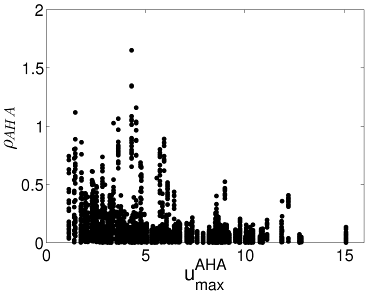

(b)

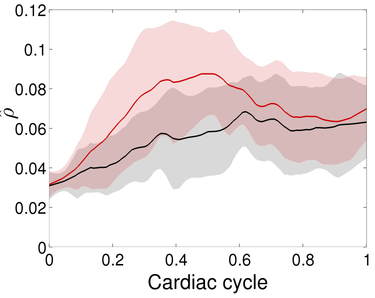

(c)

Case	V1	V2	V3	V4	V5	P1	P2	P3
				Unprocessed data				
$\rho \pm$ std	0.07 ± 0.02	0.14 ± 0.03	0.15 ± 0.03	0.11 ± 0.03	0.13 ± 0.04	0.14 ± 0.04	0.09 ± 0.02	0.16 ± 0.06
$\hat{\rho} \pm$ std	0.03 ± 0.009	0.07 ± 0.02	0.07 ± 0.02	0.05 ± 0.01	0.05 ± 0.02	0.07 ± 0.02	0.04 ± 0.006	0.06 ± 0.02
				Processed data				
$\rho \pm$ std	0.12 ± 0.04	0.20 ± 0.08	0.22 ± 0.09	0.14 ± 0.04	0.17 ± 0.05	0.16 ± 0.06	0.14 ± 0.05	0.18 ± 0.06
$\hat{\rho} \pm$ std	0.05 ± 0.02	0.08 ± 0.02	0.09 ± 0.03	0.06 ± 0.02	0.06 ± 0.02	0.08 ± 0.03	0.05 ± 0.01	0.06 ± 0.02

(d)

Figure S3 (a) Average tracking error over all cases, assessed using the error metric in Eq. S3. The error compares IRTK extracted motion (black) and processed motion (red) against manually tracked motion. The shaded regions present the standard deviation of the error between cases. (b) Error of every landmark at every timestep, against the maximum displacement of the landmark through the cycle (manual tracking). (c) Average tracking error over all cases, assessed using the error metric in Eq. S4. (d) Tracking error for each case, averaged over the cardiac cycle. Both processed and unprocessed data are considered, and the error is assessed using ρ and $\hat{\rho}$.

avoiding the requirement that precision is higher in regions with little to no motion. Tracking errors using $\hat{\rho}$ for both the unprocessed and processed extracted motion are presented in Figure S3c. Using $\hat{\rho}$ also reduced the discrepancy between operators to 0.04 ± 0.007. Additionally, average tracking errors over the cardiac cycle for all cases are presented in Figure S3d, using both error metrics.

While the processed extracted motion presented higher tracking errors than the unprocessed IRTK extracted motion, the errors remain comparable to the tracking errors reported in Shi *et al.*[1]. Moreover, examining errors using the revised error metric $\hat{\rho}$ (Figures S3c, S3d), the tracking errors of the unprocessed and unprocessed data are more consistent. While the processed motion results in slight increases in error, it also enables physiological radial thickening and wall volumes through the cardiac cycle (see Table S1).

S3 Computation of clinical metrics

Motion extracted from TMRI and processed as described in section S1 was utilised for the computation of typical clinical metrics (Table 1). End-diastolic and end-systolic volumes were computed from the respective deformed meshes of the processed data. Cavity volume was computed following the approach presented in Asner *et al.*[2,3]. Stroke volume and ejection fraction were calculated using the cavity volumes ($SV = EDV - ESV$, $EF = \frac{EDV-ESV}{EDV}$). Wall thickness at end diastole and end systole was approximated by the ratio of the wall volume over the mean surface area of endocardial and epicardial surfaces. The long-axis length was computed as the maximum distance of epicardial nodes to the centre point of the base. The short-axis dimension was computed as twice the mean distance of epicardial nodes to the long-axis.

S4 Parameter identifiability and model accuracy for *in vivo* models

	$\theta = 50°$		$\theta = 60°$		$\theta = 70°$	
**Case**	NT BC	RV BC	NT BC	RV BC	NT BC	RV BC
**V1**	0.1	0.15	0.03	0.05	0.02	0.03
**V2**	0.6	0.5	0.4	0.3	0.3	0.2
**V3**	0.05	0.1	0.02	0.03	0.02	0.02
**V4**	0.4	0.3	0.05	0.15	0.05	0.1
**V5**	0.3	0.3	0.1	0.2	0.05	0.1
**P1**	1.2	0.3	1.1	0.2	0.9	0.15
**P2**	0.9	0.7	0.5	0.5	0.2	0.3
**P3**	0.3	0.3	0.1	0.1	0.02	0.05

Table S2 Parameter ratio γ estimates for the volunteer and patient cases. Three different fibre distributions were considered, with a maximum angle of $\theta = 50°$, $\theta = 60°$ and $\theta = 70°$. Two epicardial boundary conditions were analysed, a no-traction (*NT BC*) and an RV-epicardial (*RV BC*) boundary condition.

This section examines parameter identifiability and model fidelity for the personalised models created for four volunteers (V1-V4) and three patients (P1-P3). Patient-specific models were developed following the pipeline described in sections 2.1 and 2.2. The effect of these model assumptions on the obtained parameter ratio is presented in Table S2. Additionally, the influence of the assumed fibre distribution and epicardial boundary condition on model accuracy and parameter identifiability is summarised in Figures S4 and S5.

Based on Figures S4 and S5 as well as the analysis in section 3.1 the combination of the RV BC and a fibre distribution with $\theta = 50°$ consistently resulted in lower errors. Additional assessment of model fidelity for these simulations was provided through the nodal distance between model and data (Table S3). The average nodal model error was $\sim 2mm$ and the relative errors were between 17% and 32%. These errors suggest satisfactory model accuracy.

**Case**	$e_m \pm$ std	$e_b \pm$ std	$e_{rv} \pm$ std	$e_e \pm$ std	$e_{rem} \pm$ std	$u_d \pm$ std
**V1**	1.35 ± 0.63	0.13 ± 0.11	1.20 ± 0.57	1.29 ± 0.50	1.39 ± 0.63	7.93 ± 2.89
**V2**	2.75 ± 1.50	0.23 ± 0.24	1.55 ± 0.54	2.94 ± 1.16	2.76 ± 0.98	8.40 ± 3.04
**V3**	2.10 ± 1.11	0.24 ± 0.17	1.81 ± 0.93	2.36 ± 1.19	2.09 ± 1.03	10.36 ± 4.51
**V4**	2.34 ± 1.07	0.22 ± 0.21	1.63 ± 0.69	2.88 ± 1.12	2.25 ± 0.93	8.86 ± 3.43
**V5**	1.69 ± 0.97	0.27 ± 0.22	1.61 ± 0.87	1.78 ± 0.97	1.75 ± 0.94	8.31 ± 3.52
**P1**	2.49 ± 1.27	0.11 ± 0.13	0.96 ± 0.43	2.69 ± 1.34	2.51 ± 1.19	7.88 ± 2.59
**P2**	1.90 ± 0.82	0.18 ± 0.19	1.29 ± 0.68	1.92 ± 0.82	1.94 ± 0.78	7.98 ± 3.08
**P3**	1.39 ± 0.76	0.11 ± 0.09	0.81 ± 0.34	1.56 ± 0.78	1.39 ± 0.73	7.11 ± 2.84

Table S3 Average and standard deviation of the magnitude of nodal displacement error between model and data at end-diastole in mm. Model error in this case is assessed over the entire domain (e_m), the basal surface (e_b), the RV attachment region (e_{rv}), the endocardial surface (e_e), and the remainder of the domain (e_{rem}). Also presented is the average and standard deviation of the data displacement (u_d) for each case, in mm.

Nodal model error was also computed over different regions of the LV mesh domain (Table S3). The relaxed basal boundary condition employed enabled excellent agreement ($< 0.3mm$) between model and data at the base. Low errors were also observed on the RV attachment region that was constrained to deform following the data. While low, the errors on the RV attachment region were non-negligible due to the moderately high value used for the relaxation parameter in Eq. 6. The value was selected to enable a good

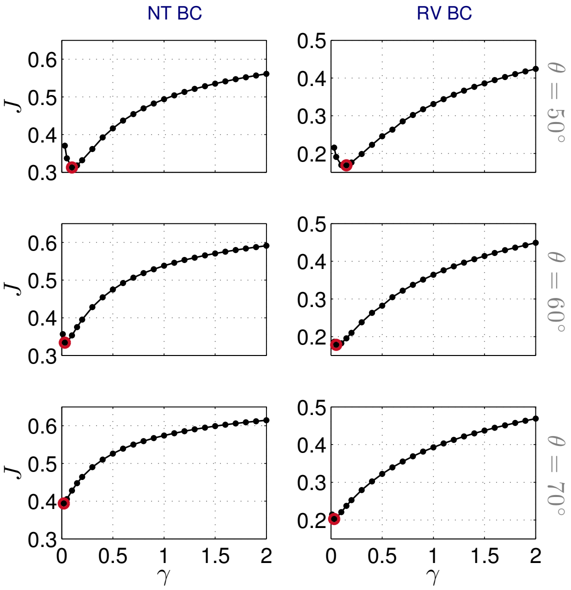

(a)

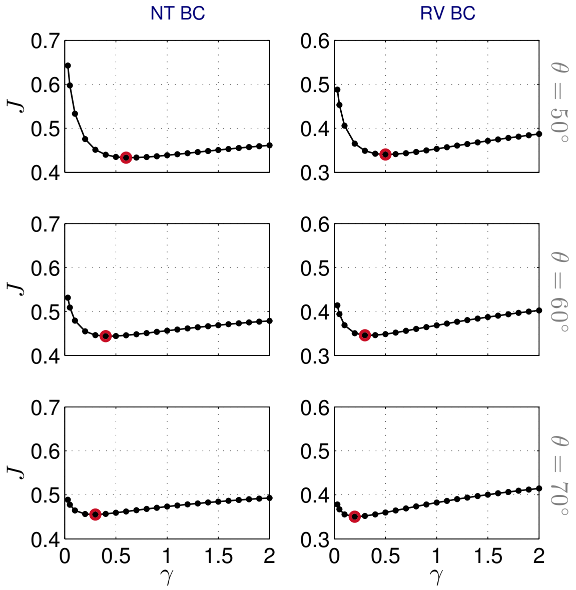

(b)

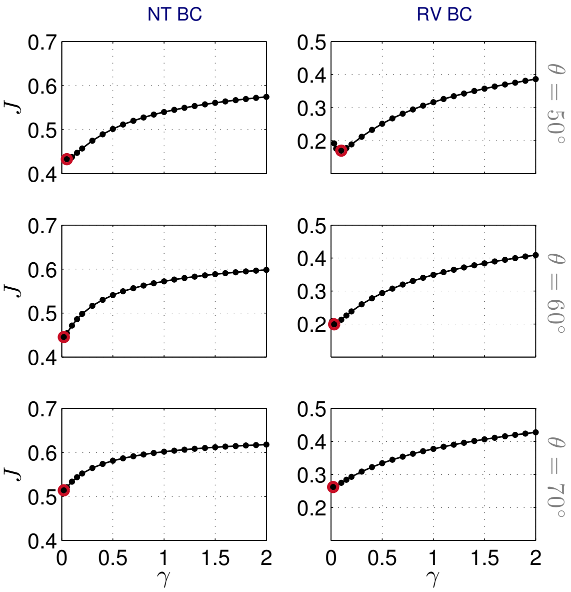

(c)

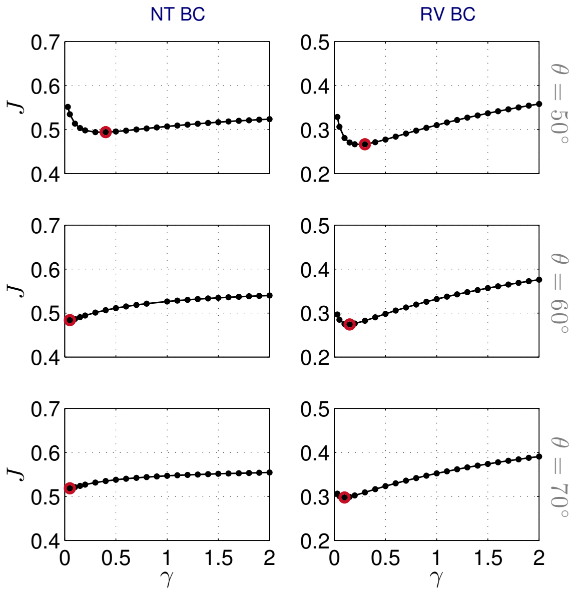

(d)

Figure S4 $\mathcal{J}$ over the parameter ratio γ for **(a)** V1, **(b)** V2, **(c)** V3 and **(d)** V4. Three different fibre distributions were considered, with a maximum angle of $\theta = 50°$, $\theta = 60°$ and $\theta = 70°$. Two epicardial boundary conditions were analysed, a no-traction (*NT BC*) and an RV-epicardial (*RV BC*) boundary condition.

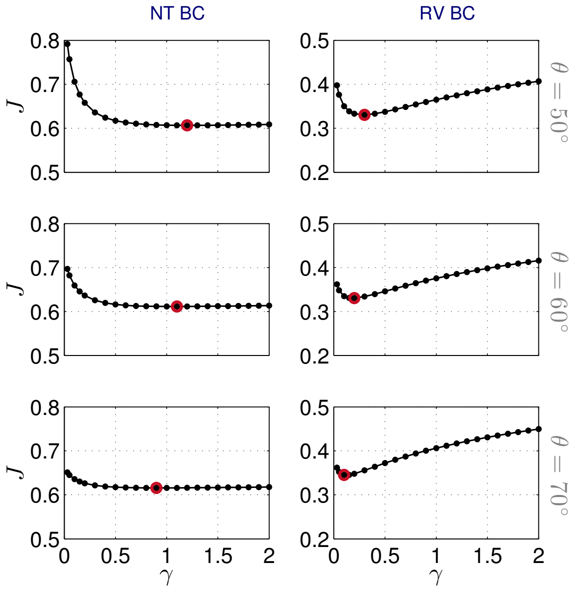

(a)

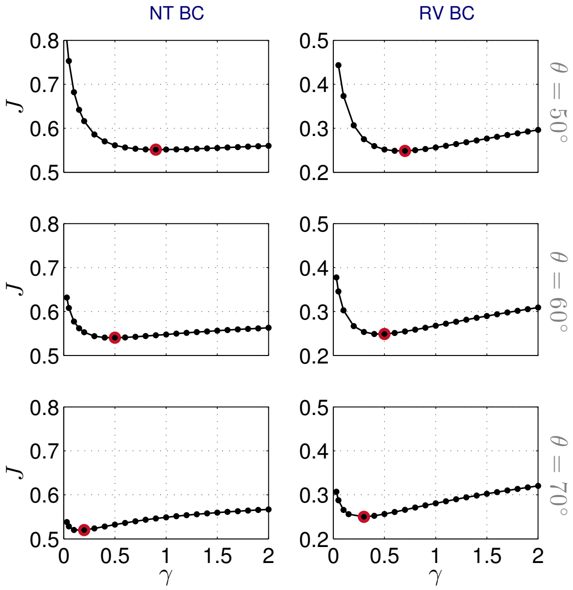

(b)

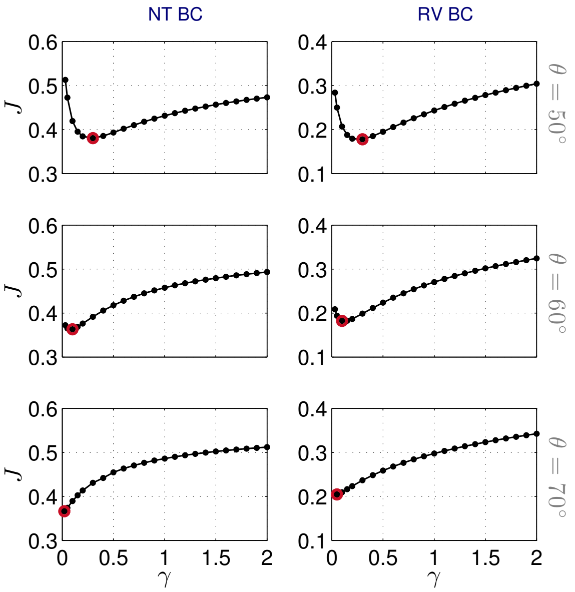

(c)

Figure S5 $\mathcal{J}$ over the parameter ratio γ for **(a)** P1, **(b)** P2 and **(c)** P3. Three different fibre distributions were considered, with a maximum angle of $\theta = 50°$, $\theta = 60°$ and $\theta = 70°$. Two epicardial boundary conditions were analysed, a no-traction ($NT\ BC$) and an RV-epicardial ($RV\ BC$) boundary condition.

agreement with the data without causing pressure peaks. The displacement errors were higher on the endocardial surface ($< 3mm$) where only the cavity volume was prescribed. Reasonable errors ($< 3mm$) were observed in the mesh region which was not driven by the data, indicating the ability of the model to reproduce cardiac behaviour.

Additionally, the effect of the data frame assumed as reference domain was assessed on all volunteer and patient cases. Following the description of section 3.1, in Figure S6 parameter identifiability and parameter estimates were examined using three different data-frames (end-systolic, second and fourth after end-systolic frames), for V2-V5 and P2-P3.

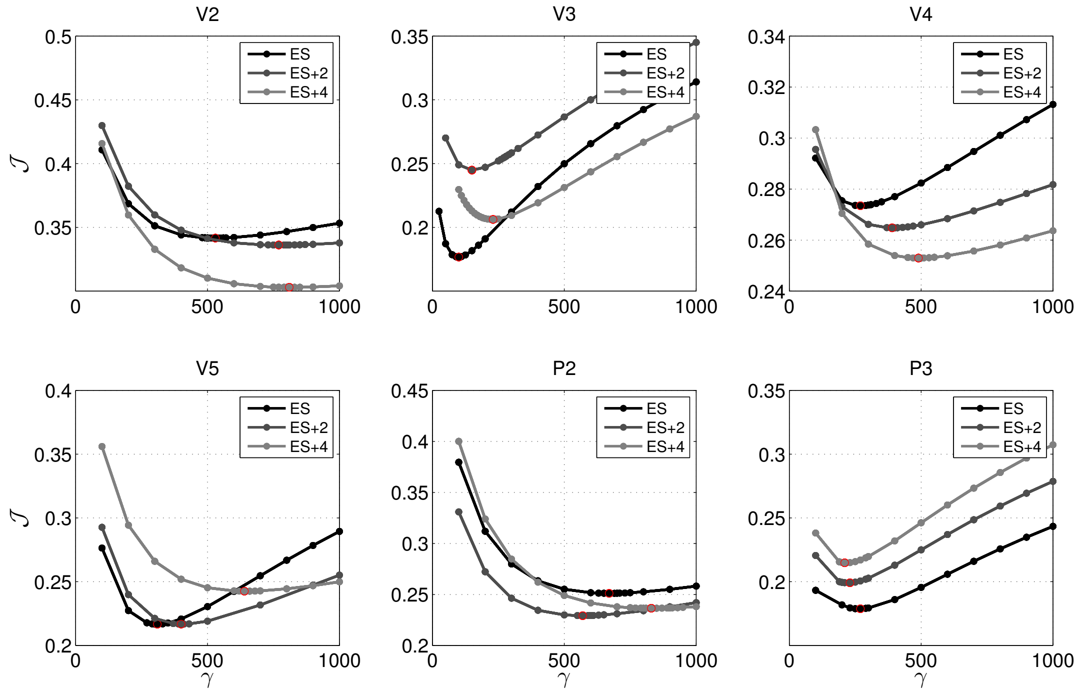

Figure S6 Objective function $\mathcal{J}$ over the parameter ratio γ, with three different data frames assumed as the reference geometry.

S5 Regional strain analysis

This section provides an additional means of assessing model accuracy through the evaluation of regional strain distributions in both data and models. Analysis of regional cardiac behaviour derived from strains might elucidate differences between DCM and volunteer cases which could not be derived from global metrics. To this end, each personalised geometry

was divided in 16 regions according to the American Heart Association (AHA) recommendations[2]. The apex was not included as a 17th region due to difficulties in accurately identifying the apical location.

Based on the Lagrange-Green definition, $\boldsymbol{E} = \frac{1}{2}(\boldsymbol{F}^T\boldsymbol{F} - \boldsymbol{I})$, strain $\boldsymbol{E}$ is dependent on spatial derivatives of data-derived displacement and is thus very sensitive to noise. In order to circumvent this data sensitivity, an alternative way of computing strain was employed. Considering the fundamental definition of the deformation gradient ($d\boldsymbol{x} = \boldsymbol{F}d\boldsymbol{X}$), $\boldsymbol{F}$ maps infinitesimal vectors $d\boldsymbol{X}$ in the undeformed domain to their corresponding vectors $d\boldsymbol{x}$ in the deformed domain. Instead of computing $\boldsymbol{F}$ at every node in the geometry, an approximate value $\bar{\boldsymbol{F}}$ per AHA region was estimated. Specifically, for each mesh node of the AHA region, the "infinitesimal" vectors $d\bar{\boldsymbol{x}}$ and $d\bar{\boldsymbol{X}}$ were computed with respect to the centroid of the region in the deformed and undeformed domain, respectively. $\bar{\boldsymbol{F}}$ for every AHA region was then computed as the tensor that satisfied $d\bar{\boldsymbol{x}} = \bar{\boldsymbol{F}}d\bar{\boldsymbol{X}}$ for all nodes k in the region, in the least-squares sense:

$$\sum_k ||d\bar{\boldsymbol{x}}_k - \bar{\boldsymbol{F}}d\bar{\boldsymbol{X}}_k||^2 = \min_{\boldsymbol{A}\in\mathbb{R}^{3\times3}} \sum_k ||d\bar{\boldsymbol{x}}_k - \boldsymbol{A}d\bar{\boldsymbol{X}}_k||^2. \tag{S5}$$

Strain was subsequently computed using the approximated deformation gradient per region as

$$\bar{\boldsymbol{E}} = \frac{1}{2}(\bar{\boldsymbol{F}}^T\bar{\boldsymbol{F}} - \boldsymbol{I}). \tag{S6}$$

The use of definition S6 for strain avoids bias introduced by noise-sensitive derivative estimates. Combined with the use of mean quantities per AHA region, this strain definition should reduce noise sensitivity, providing physiological strains.

Using this definition, strain was computed for all AHA regions and reported values correspond to strains at end diastole with respect to end systole. Strains in the radial ($E_{rr} = \bar{\boldsymbol{E}}\boldsymbol{r}\cdot\boldsymbol{r}$), longitudinal ($E_{ll} = \bar{\boldsymbol{E}}\boldsymbol{l}\cdot\boldsymbol{l}$) and circumferential ($E_{\theta\theta} = \bar{\boldsymbol{E}}\boldsymbol{\theta}\cdot\boldsymbol{\theta}$) directions were also computed for each region, with $\boldsymbol{r}$, $\boldsymbol{l}$ and $\boldsymbol{\theta}$ the unit vectors in the radial, long-axis and circumferential directions, respectively. The spectral norm of the strain tensor was computed as well, as the maximum strain eigenvalue ($||\bar{\boldsymbol{E}}||_2 = \lambda_{max}(\bar{\boldsymbol{E}})$), providing a global metric.

Initially, average values of strain metrics over all regions were considered, to provide

**Case**	E_{rr}	E_{ll}	$E_{\theta\theta}$	$\|\|\bar{\boldsymbol{E}}\|\|_2$
Volunteers				
**V1**	-0.13	0.19	0.17	0.27
**V2**	-0.13	0.13	0.19	0.28
**V3**	-0.18	0.26	0.18	0.34
**V4**	-0.14	0.14	0.17	0.30
**V5**	-0.15	0.19	0.19	0.29
**Mean**	-0.14	0.18	0.18	0.29
**SD**	0.02	0.05	0.01	0.03
DCM Patients				
**P1**	-0.13	0.16	0.14	0.24
**P2**	-0.12	0.15	0.17	0.25
**P3**	-0.12	0.14	0.15	0.24
**Mean**	-0.12	0.15	0.15	0.24
**SD**	0.006	0.01	0.01	0.003

Table S4 Average data strain at end diastole with respect to end systole for all volunteer and patient cases. Strain in the radial (E_{rr}), longitudinal (E_{ll}) and circumferential ($E_{\theta\theta}$) directions along with strain norm ($||\bar{\boldsymbol{E}}||_2$) are presented.

overall deformation characteristics (Table S4) and enable direct comparisons between the volunteer and patient groups. As indicated by the decrease in $||\bar{\boldsymbol{E}}||_2$ in the DCM group ($p = 0.02$), global strain was decreased in DCM hearts. In fact, strain in the radial and circumferential directions was notably reduced in the patient group ($p < 0.1$ for both E_{rr} and $E_{\theta\theta}$). Decrease was also observed in longitudinal strain, even though not statistically significant.

Strain per AHA region was also computed for the personalised models, to enable regional comparisons between data and model. Data and model strains per AHA region are presented for a representative example (V3) in Figure S7. Notable similarities can be observed between spatial distributions of data and model strains. Despite differences in magnitude, the regions of maximum and minimum strains are – in most cases – consistent between model and data. The similarity in strain distributions suggests that the personalised models reasonably reflect the true data-derived strains.

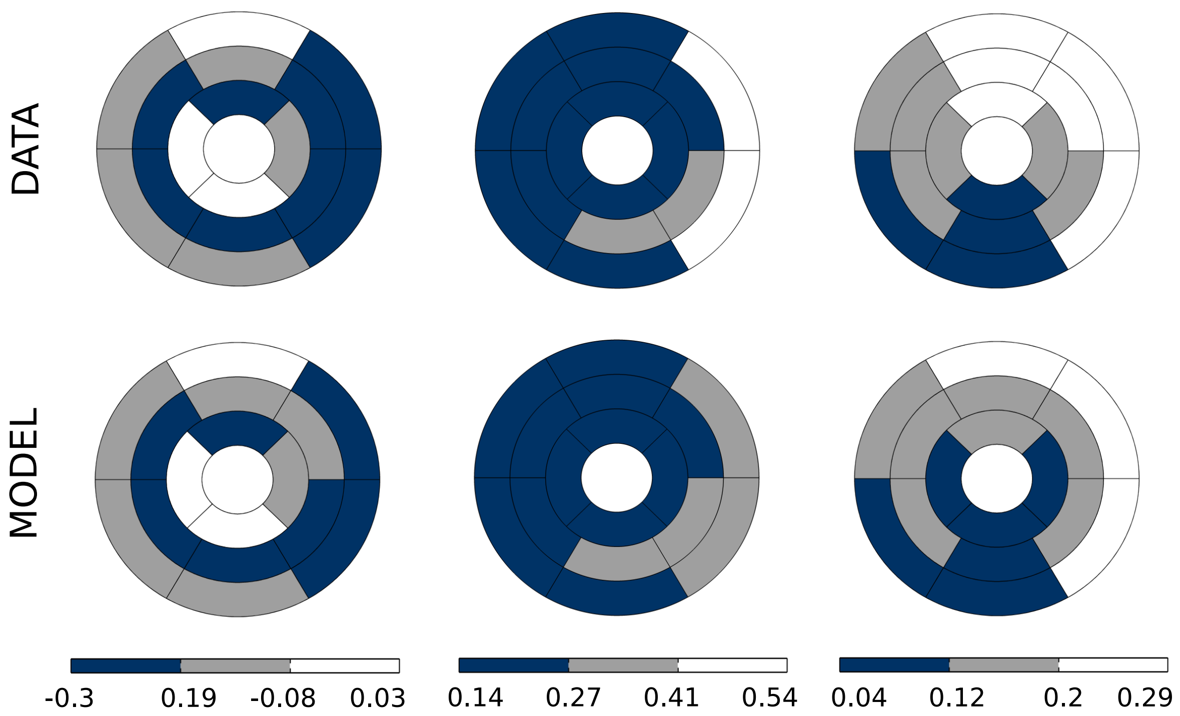

Figure S7 Strain for V3 at end diastole per *AHA* region in **(left)** radial, **(middle)** longitudinal and **(right)** circumferential directions. Strain is computed for data **(top row)** and model **(bottom row)** with respect to end systole.

References

1. Shi, W., X. Zhuang, H. Wang, S. Duckett, D. Luong, C. Tobon-Gomez, K. Tung, P. Edwards, K. Rhode, R. Razavi, S. Ourselin, and D. Rueckert. A comprehensive cardiac motion estimation framework using both untagged and 3-D tagged MR images based on nonrigid registration. IEEE Trans. Med. Imag. 31:1263–75, 2012.

2. Cerqueira, M. N. Weissman, V. Dilsizian, A. Jacobs, S. Kaul, W. Laskey, D. Pennell, J. Rumberger, T. Ryan, , M. Verani. Standardized Myocardial Segmentation and Nomenclature for Tomographic Imaging of the Heart Journal of Cardiovascular Magnetic Resonance. 203–210, 2002
